# Evaluation of Veterinary-Specific Interpretive Criteria for Susceptibility Testing of Streptococcus equi Subspecies with Trimethoprim-Sulfamethoxazole and Trimethoprim-Sulfadiazine

**DOI:** 10.1128/JCM.01610-16

**Published:** 2016-12-28

**Authors:** Carmen Sadaka, Theo Kanellos, Luca Guardabassi, Joseph Boucher, Jeffrey L. Watts

**Affiliations:** a Zoetis Global Therapeutics Research, Kalamazoo, Michigan, USA; b Zoetis Inc., Paris, France; c Department of Veterinary Disease Biology, Faculty of Health and Medical Sciences, University of Copenhagen, Frederiksberg, Denmark; d Department of Biomedical Sciences, Ross University School of Veterinary Medicine, Basseterre, St. Kitts and Nevis, West Indies; University of Tennessee

**Keywords:** breakpoints, horses, interpretive criteria, potentiated sulfonamides, strangles, veterinary microbiology

## Abstract

Antimicrobial susceptibility test results for trimethoprim-sulfadiazine with Streptococcus equi subspecies are interpreted based on human data for trimethoprim-sulfamethoxazole. The veterinary-specific data generated in this study support a single breakpoint for testing trimethoprim-sulfamethoxazole and/or trimethoprim-sulfadiazine with S. equi. This study indicates trimethoprim-sulfamethoxazole as an acceptable surrogate for trimethoprim-sulfadiazine with S. equi.

## TEXT

Trimethoprim-sulfadiazine (SXD) is one of the antimicrobial agents prescribed for the treatment of strangles and other equine infections caused by Streptococcus equi subsp. equi and Streptococcus equi subsp. zooepidemicus (1). The CLSI veterinary standard (VET01-A4) lists breakpoints for trimethoprim-sulfamethoxazole (SXT) for Streptococcus pneumoniae species, but not for SXD and beta-hemolytic streptococci (2). Additionally, these breakpoints are based on human-derived data (3). Therefore, the interpretation of antimicrobial susceptibility test (AST) results in compliance with CLSI for S. equi is based on the clinical breakpoint for Streptococcus pneumoniae with SXT used in human medicine (susceptible [S] ≤ 0.5/9.5 μg/ml, intermediate [I] = 1/19 to 2/38 μg/ml, resistant [R] ≥ 4/76 μg/ml and S ≥ 19 mm, I = 16 to 18 mm, R ≤ 15 mm) (2, 3). This breakpoint may not be appropriate for equine isolates due to the pharmacokinetic/pharmacodynamic (PK/PD) differences of the trimethoprim-sulfonamide combinations between horses and humans and because of the PK/PD variability of different potentiated sulfonamide combinations on different bacterial species, sometimes even within the same genus (4). There is limited information on the susceptibility of S. equi subspecies to both SXT and SXD. Moreover, SXT is used as a class representative for susceptibility testing of SXD by veterinary diagnostic laboratories, but this use has not been validated specifically for equine isolates (2). This study had four goals: (i) determine if SXT is an acceptable surrogate drug for SXD when testing equine streptococcal isolates; (ii) assess the *in vitro* activity of SXD and SXT against clinical isolates of S. equi; (iii) evaluate the synergistic ratios for SXD and SXT with equine S. equi; and (iv) evaluate whether the human breakpoints are appropriate for use with equine isolates.

(This study was presented in part at the 57th Annual Conference of the American Association of Veterinary Laboratory Diagnosticians, Kansas City, MO, 16 to 22 October 2014 [5] and the International Conference on One Health Antimicrobial Resistance, Copenhagen, Denmark, 30 September to 2 October 2015 [6]).

A total of 270 S. equi isolates divided equally between S. equi subsp. equi (*n* = 135) and S. equi subsp. zooepidemicus (*n* = 135) isolated from clinical cases of strangles or equine infectious respiratory disease were used in the study. All isolates were collected from laboratories participating in the Zoetis Global Therapeutics Research surveillance program (2002 to 2009) from different geographic locations (USA [*n* = 156], Canada [*n* = 94], and Europe [*n* = 20]).

Minimal inhibitory concentration (MIC) as determined by the broth microdilution procedure and agar disk diffusion tests were conducted (*n* = 270) as previously described (2, 7). SXT disks (1.25/23.75 μg) (Oxoid Ltd.) were used in the disk diffusion assay. MICs were determined for sulfadiazine (SDZ), sulfamethoxazole (SMX), trimethoprim (TMP), and their corresponding TMP-sulfa combinations (1:19 ratio) (Sigma-Aldrich). Quality control was conducted using CLSI-recommended quality control strains (for MIC assay, Enterococcus faecalis American Type Culture Collection [ATCC] 29212, Streptococcus pneumoniae ATCC 49619, and Staphylococcus aureus ATCC 29213; for disk diffusion assay, E. faecalis ATCC 29212, S. pneumoniae ATCC 49619, S. aureus ATCC 25923, and Escherichia coli ATCC 25922) and antimicrobial agents (SXT disks, amoxicillin-clavulanic acid combination [2:1], SXT combination [1:19], and ampicillin). Cation-adjusted Mueller-Hinton broth (CA-MHB) and cation-adjusted Mueller-Hinton agar (CA-MHA) (Fisher Scientific) were prepared in-house as per the instructions of the manufacturer. Thymidine phosphorylase (200 U/liter) (Sigma-Aldrich) was added to all media prior to use to make sure that the media were as thymidine-free and thymine-free as possible. Lysed horse blood (2 to 5%) (Hema Resource) was added to CA-MHB, and sheep blood (5%) (Cleveland Scientific) was added to CA-MHA used for Streptococcus species only.

Fractional inhibitory concentration (FIC) indices were determined using the checkerboard technique to evaluate optimal drug ratios for synergy (FIC index of ≤0.5) on a selection of isolates (10 S. equi subsp. zooepidemicus isolates and 10 S. equi subsp. equi isolates) (8). These isolates were chosen on the basis of very poor activity of individual sulfonamides (MIC > 2,084 μg/ml). Quality control included testing for synergy (amoxicillin and clavulanic acid) and antagonism (sparfloxacin and chloramphenicol) reproducibility in the reference S. aureus strain ATCC 29213 (9, 10). FIC indices were determined by using the method described by Krogstad and Moellering (8).

**FIG 1:**
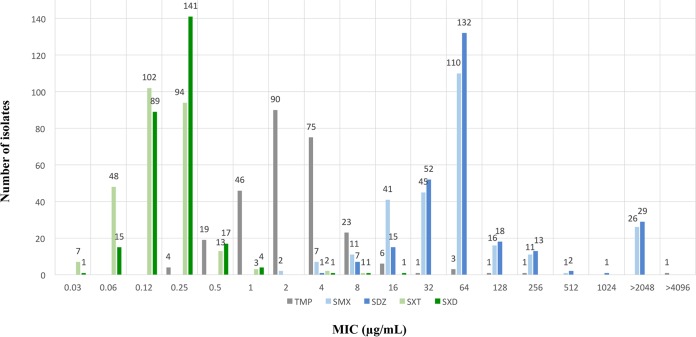
Histograms of MIC distributions for TMP, SMX, SDZ, SXT, and SXD for 270 S. equi isolates. MIC values for SXT and SXD are plotted based on the TMP concentration in each combination antimicrobial (TMP-to-sulfonamide combination ratio 1:19). The concentration ranges for SXT and SXD tested are 0.03/0.6 to 32/608 μg/ml.

The MIC results are summarized in Fig. 1. Poor activity was recorded against individual drugs (MIC_90_ values of 8, 256, and >2,048 μg/ml for TMP, SMX, and SDZ, respectively). MICs of SXT (MIC_90_ of 0.25/4.8 μg/ml) and SXD (MIC_90_ of 0.25/4.8 μg/ml) at a ratio 1:19 were recorded for all tested isolates. A bimodal distribution was noticed with an epidemiological cutoff value of ≤1/19 μg/ml for wild type for both SXT and SXD (Fig. 1). Test error rates were determined by the standard error rate-bounded method (11, 12) using the R statistical computing program (Frisbee Sailing 2013-09-25 version 3.0.2) to plot scattergrams.

**FIG 2:**
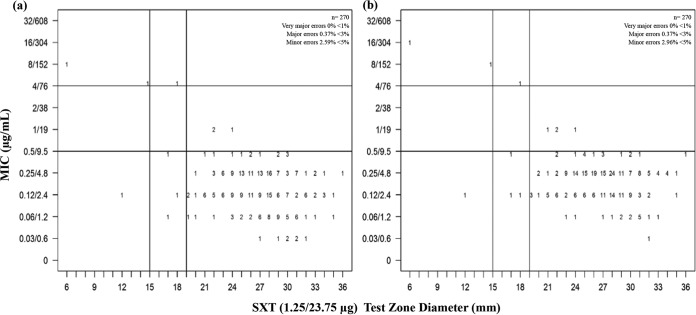
Scattergrams of (a) SXT MIC and (b) SXD MIC versus SXT (1.25/23.75 μg) disk diffusion test zone diameter (in millimeters) for 270 S. equi isolates. Numbers represent the numbers of isolates at each MIC/test zone diameter pair. The horizontal and vertical lines represent MIC and disk diffusion cutoffs, respectively.

**FIG 3:**
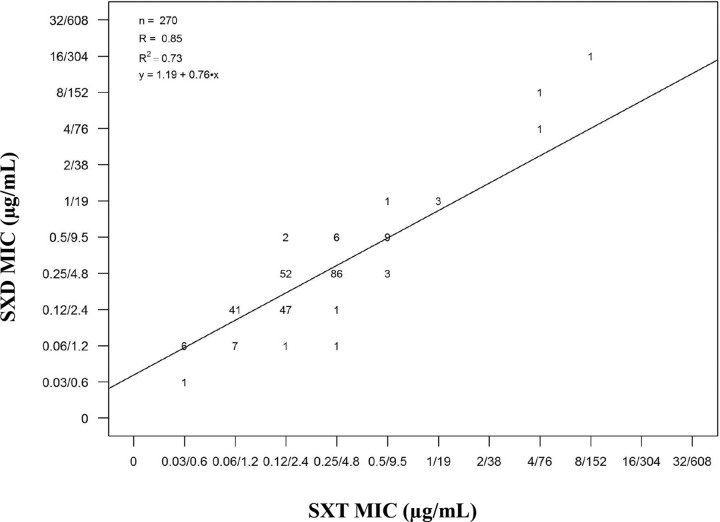
Scattergram of SXD versus SXT MICs (in micrograms per milliliter) for the 270 S. equi isolates tested. The scattergram also shows the linear regression with *R* = 0.85. Numbers represent the numbers of isolates at each SXD/SXT MIC pair.

The disk diffusion method remains one of the most frequently employed AST method (13). One of the challenges of this method with potentiated sulfonamides is testing for SXD susceptibility using SXT disks without knowing whether the latter is representative (2). Veterinary-specific epidemiological cutoffs were set for both subspecies. Based on the MIC (S ≤ 0.5/9.5 μg/ml; I = 1/19 to 2/38 μg/ml, and R ≥ 4/76 μg/ml) and test zone diameter (S ≥ 19 mm; I = 16 to 18 mm, and R ≤ 15 mm) cutoffs, the discrepancy rates were within the acceptable range (very major errors ≤ 1%, major errors ≤ 3.5%, and minor errors ≤ 5%) (Fig. 2). SXT disks are therefore good surrogates for AST of potentiated sulfonamides. Moreover, the scattergram for SXD MICs versus SXT MICs showed that SXT is a suitable representative for SXD (R = 0.85) (Fig. 3), with the consideration that SXT may be more active by at least one twofold dilution (40.74% [110/270]) (data not shown).

The FIC indices (for S. equi subsp. equi and S. equi subsp. zooepidemicus, SXT FIC_index average_ values of 0.333 and 0.335, respectively, and SXD FIC_index average_ values of 0.321 and 0.381, respectively) showed synergy at a wide range of TMP-to-sulfonamide ratios and concentrations (1:1 to 1:256) (see Table S1 in the supplemental material). The current findings are in accordance with those from a similar study conducted on 59 different equine pathogens (5). Additionally, the results comply with those from a susceptibility study by the agar dilution method where TMP and nine sulfonamide combinations exhibited synergy against equine Salmonella enterica serovars (*n* = 62) at all tested TMP-to-sulfonamide ratios (1:1 to 1:160) for all tested sulfonamides, including SDZ but not SMX (synergy recorded at all ratios except at the 1:1 ratio) (14). In human medicine, potentiated sulfonamides are administered at a 1:5 TMP-to-sulfa ratio to achieve the 1:20 plasma ratio required for clinical efficacy. It has been shown in equine practice that potentiated sulfonamides achieve the 1:20 plasma ratio and can be regarded to as therapeutically effective for susceptible S. equi when administered at 5 mg of TMP/kg of body weight and 25 mg of SDZ/kg (1:5 ratio) with a dosing interval of 12 h (15–19). The results support the available literature, since the FIC indices showed synergy at a wide range of TMP-to-sulfonamide ratios and concentrations.

Overall, the data indicated low resistance figures (≤2.2%) for both subspecies regardless of the AST method used (data not shown). Similarly, Schwarz et al.(20) have also reported low resistance figures (0%). However, high resistance values have been reported in the literature (25.9% and 42% for S. equi subsp. equi and 45.5% and 61.1% for S. equi subsp. zooepidemicus [21, 22]). While the reasons for the discrepancies in resistance levels are not known, test methodologies could account for some of these differences, as it is well established that thymidine levels in the test media can diminish the activity of sulfas resulting in false resistance (23). We recommend following the CLSI guidelines to determine whether the conditions of the growth medium are satisfactory and ensure that excessive thymidine levels are not present in the test medium (2).

On the basis of the data generated in this study, we propose that the current CLSI interpretive criteria for S. pneumoniae (MIC cutoffs of S ≤ 0.5/9.5 μg/ml, I = 1/19 to 2/38 μg/ml, and R ≥ 4/76 μg/ml and test zone diameter cutoffs of S ≥ 19 mm, I = 16 to 18 mm, and R ≤ 15 mm) are suitable for testing equine S. equi. In the absence of resistant strains, we recommend veterinary-specific interpretive criteria for susceptible strains only for testing SXT and SXD with equine streptococcal isolates (MIC cutoff of S ≤ 0.5/9.5 μg/ml and test zone diameter cutoff of S ≥ 19 mm).

In summary, the data generated in this study indicate that SXT is an appropriate class representative for AST of SXD in S. equi subsp. The generated data are consistent with the human-derived interpretative criteria for SXT with S. pneumoniae. On the basis of the generated data, we propose a single susceptible breakpoint for testing SXT and SXD in S. equi strains: a MIC cutoff of ≤0.5/9.5 μg/ml and a zone diameter cutoff of ≥19 mm.

## Supplementary Material

Supplemental material
