# Supplementary material for: Evaluation of Veterinary-Specific Interpretive Criteria for Susceptibility Testing of Streptococcus equi Subspecies with Trimethoprim-Sulfamethoxazole and Trimethoprim-Sulfadiazine
Source: J Clin Microbiol. 2016 Dec 28;55(1):326–30. doi: 10.1128/JCM.01610-16 (PMC5228247; doi:10.1128/JCM.01610-16)
Supplement: Supplemental material [file supp_55_1_326__index.html]

Supplemental material 

# Evaluation of Veterinary-Specific Interpretive Criteria for Susceptibility Testing of Streptococcus equi Subspecies with Trimethoprim-Sulfamethoxazole and Trimethoprim-Sulfadiazine

## Supplemental material

- Supplemental file 1 -

  Fig. S1 (Histograms of MIC distributions for TMP, SMX, SDZ, SXT, and SXD for *S. equi* subsp. *equi* and *S. equi* subsp. *zooepidemicus*), S2 (Scattergrams of SXT MIC and SXD MIC versus SXT disk diffusion test zone diameter for *S. equi* subsp. *equi* isolates), and S3 (Scattergrams of SXT MIC and SXD MIC versus SXT disk diffusion test zone diameter for *S. equi* subsp. *zooepidemicus* isolates) and Table S1 (FIC indexes and FIC index averages for SXT and SXD in 10 isolates each of *S. equi* subsp. *equi* and *S. equi* subsp. *zooepidemicus*)

  PDF, 610K
