# Supplementary material for: Evaluation of Veterinary-Specific Interpretive Criteria for Susceptibility Testing of Streptococcus equi Subspecies with Trimethoprim-Sulfamethoxazole and Trimethoprim-Sulfadiazine
Source: J Clin Microbiol. 2016 Dec 28;55(1):326–30. doi: 10.1128/JCM.01610-16 (PMC5228247; doi:10.1128/JCM.01610-16)
Supplement: Supplemental material [file JCM.01610-16_zjm999095328s1.pdf]

SUPPLEMENTAL DATA

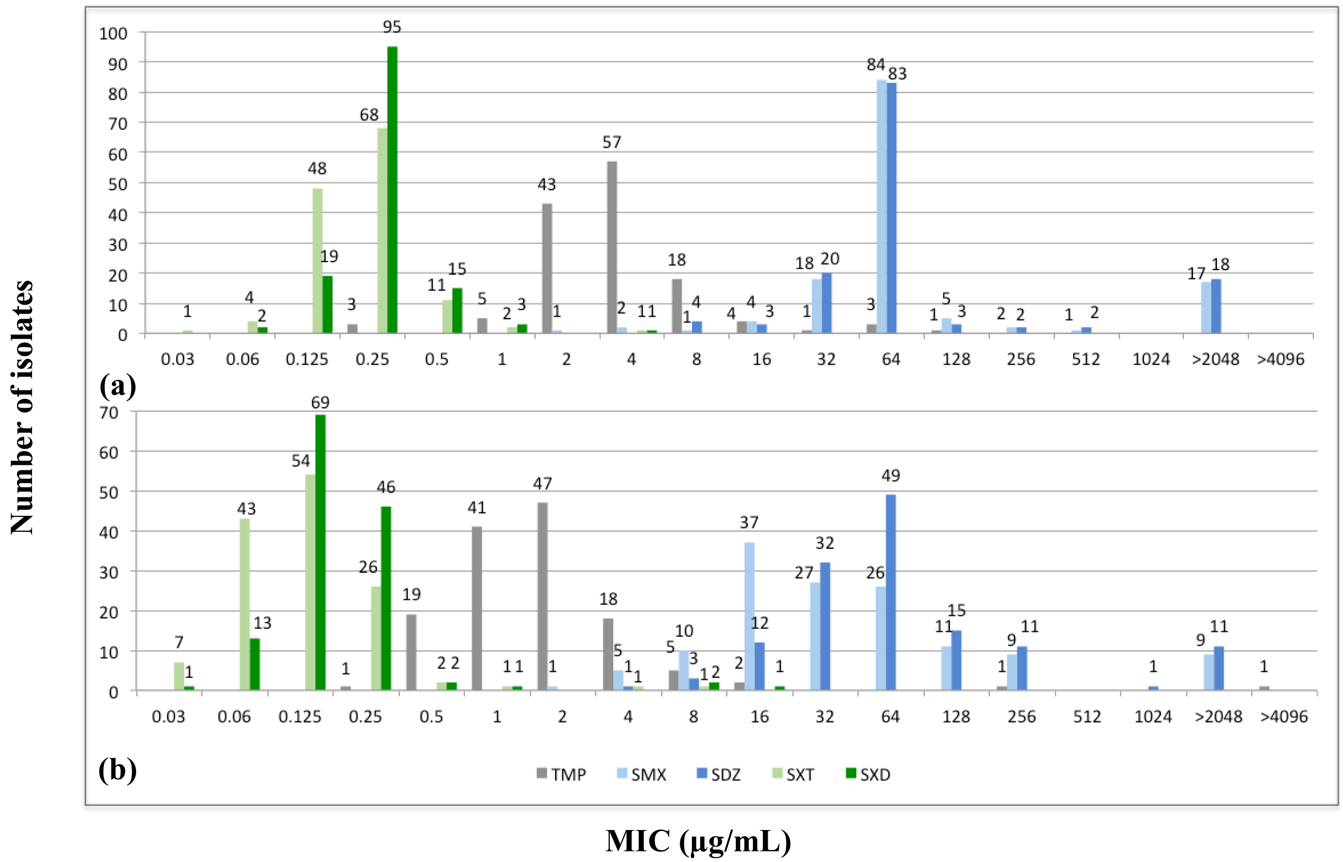

FIG 1. Histograms of MIC distributions for TMP, SMX, SDZ, SXT and SXD for (a) *S. equi* subsp. *equi* (n=135) and (b) *S. equi* subsp. *zooepidemicus* (n=135). MIC values for SXT and SXD are plotted based on the TMP concentration in each combination antimicrobial (TMP-to-sulfonamide combination ratio 1:19)**.** Tested concentration ranges for SXT and SXD are 0.03/0.6-32/608 µg/mL.

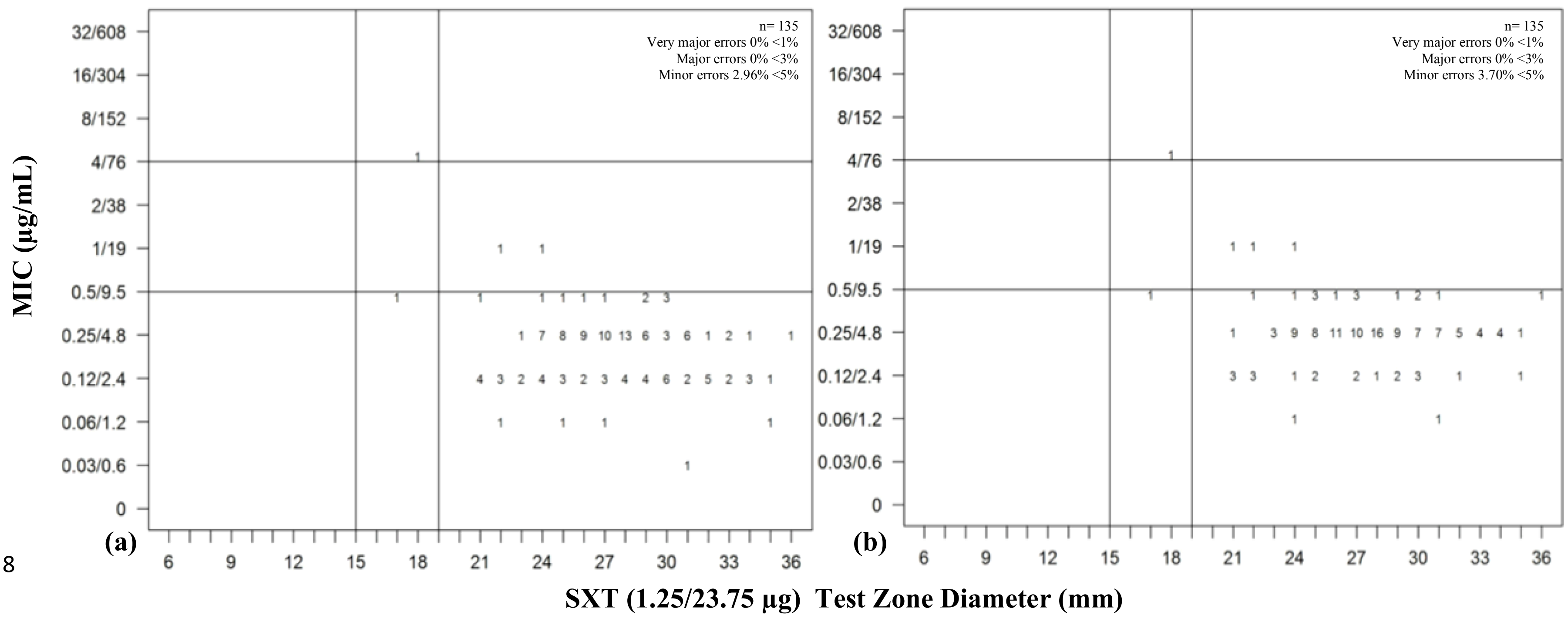

FIG 2. Scattergrams of (a) SXT MIC and (b) SXD MIC versus SXT (1.25/23.75 µg) disk diffusion test zone diameter (mm) for *S. equi* subsp. *equi* isolates (n=135). Numbers represent the numbers of isolates at each MIC/test zone diameter pair. The horizontal and vertical lines represent MIC and disk diffusion cut-offs, respectively.

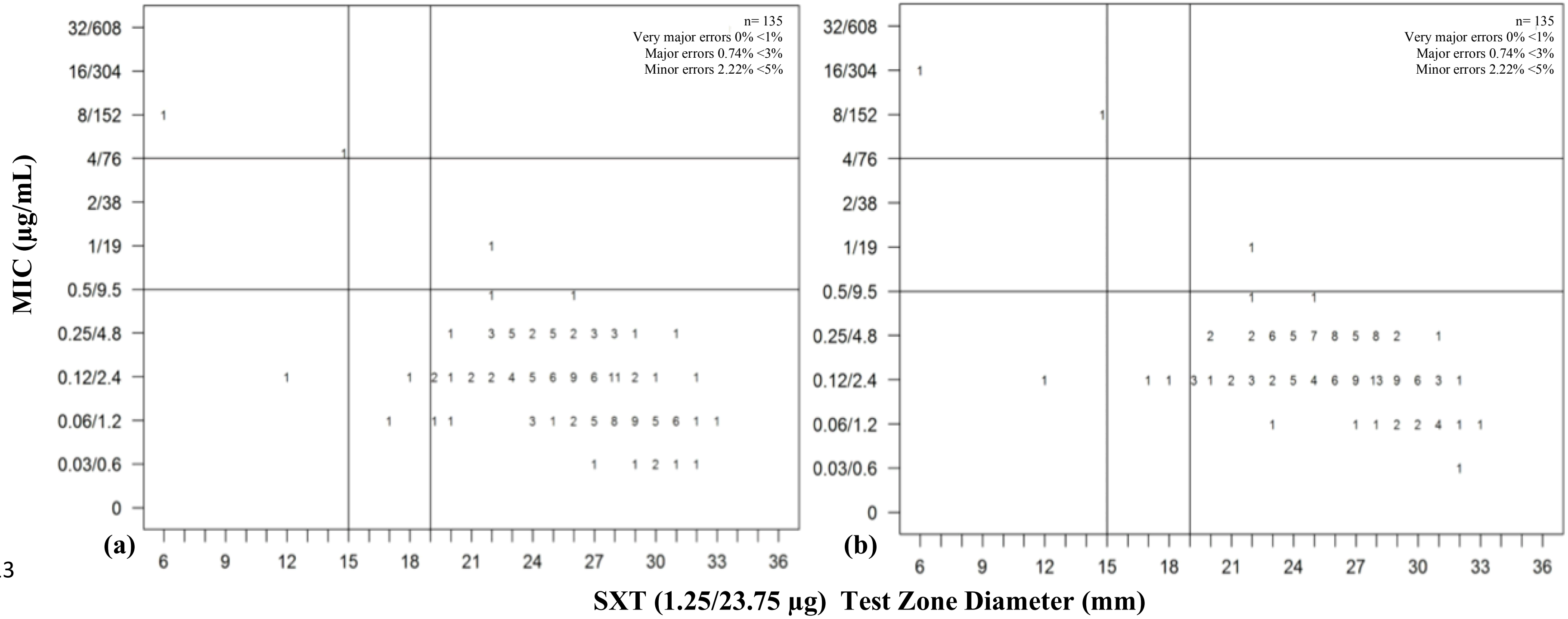

FIG 3. Scattergrams of (a) SXT MIC and (b) SXD MIC versus SXT (1.25/23.75 µg) disk diffusion test zone diameter (mm) for *S. equi* subsp. *zooepidemicus* isolates (n=135). Numbers represent the numbers of isolates at each MIC/test zone diameter pair. The horizontal and vertical lines represent MIC and disk diffusion cut-offs, respectively.

TABLE 1. FIC indexes and FIC indexes averages for SXT and SXD in 10 isolates of each *S. equi* subsp. *equi* and *S. equi* subsp. *zooepidemicus*

		FIC index				FIC index	
Strain		SXT	SXD	Strain		SXT	SXD
S. equi subp. *equi*	1	0.34	0.34	*S equi subp. zooepidemicus*	1	0.1	0.43
	2	0.34	0.32		2	0.38	0.29
	3	0.31	0.34		3	0.31	0.48
	4	0.23	0.38		4	0.31	0.32
	5	0.5	0.18		5	0.43	0.45
	6	0.29	0.35		6	0.31	0.31
	7	0.48	0.38		7	0.47	0.43
	8	0.3	0.4		8	0.35	0.46
	9	0.34	0.22		9	0.32	0.45
	10	0.2	0.3		10	0.37	0.19
FIC index average		0.333	0.321	FIC index average		0.335	0.381
